# Whole-genome resequencing of *Sorghum bicolor* and *S. bicolor* × *S. halepense* lines provides new insights for improving plant agroecological characteristics

**DOI:** 10.1038/s41598-022-09433-0

**Published:** 2022-04-01

**Authors:** Ephrem Habyarimana, Sunita Gorthy, Faheem S. Baloch, Sezai Ercisli, Gyuhwa Chung

**Affiliations:** 1 International Crops Research Institute for the Semi-Arid Tropics, Patancheru, 502 324 Telangana India; 2 Faculty of Agricultural Sciences and Technologies, Sivas University of Science and Technology, Sivas, Turkey; 3 Department of Horticulture, Faculty of Agriculture, Ataturk University, 25240 Erzurum, Turkey; 4 Department of Biotechnology, Chonnam National University, Chonnam, Republic of Korea

**Keywords:** Biotechnology, Computational biology and bioinformatics, Genetics, Molecular biology, Plant sciences

## Abstract

Sorghum (*Sorghum bicolor* L. (Moench)) is the world’s fifth economically most important cereal and is a staple particularly in the semi-arid tropics of Africa and Asia. Genetic gains in this crop can benefit from wild relatives such as *Sorghum halepense*. Genome sequences including those from this wild species can boost the study of genome-wide and intraspecific variation for dissecting the genetic basis and improving important traits in sorghum. The whole-genome resequencing carried out in this work on a panel of 172 populations of *S. bicolor* and *S. bicolor* × *S. halepense* (SbxSh) advanced lines generated a total of 567,046,841 SNPs, 91,825,474 indels, 1,532,171 SVs, and 4,973,961 CNVs. Clearly, SbxSh accumulated more variants and mutations with powerful effects on genetic differentiation. A total of 5,548 genes private to SbxSh mapped to biological process GO enrichment terms; 34 of these genes mapped to root system development (GO: 0022622). Two of the root specific genes i.e., ROOT PRIMORDIUM DEFECTIVE 1 (RPD1; GeneID: 8054879) and RETARDED ROOT GROWTH (RRG, GeneID: 8072111), were found to exert direct effect on root growth and development. This is the first report on whole-genome resequencing of a sorghum panel that includes *S. halepense* genome. Mining the private variants and genes of this wild species can provide insights capable of boosting sorghum genetic improvement, particularly the perenniality trait that is compliant with agroecological practices, sustainable agriculture, and climate change resilience.

## Introduction

Sorghum (*Sorghum bicolor* L. (Moench), 2*n* = 2*x* = 20) is the world’s fifth economically most important cereal^1^; it is a staple particularly in the semi-arid tropics of Africa and Asia, representing 6.5 million square kilometers in over 55 countries, and home to more than 2 billion people of which 600 million are considered to be poor^2^. Sorghum is now becoming popular in the food industry worldwide, due to the rise in demand for gluten-free specialty grains rich in health-promoting and food oxidative stabilizing compounds^3,4^. Indeed, sorghum grains, particularly red varieties, exhibit the highest values of total antioxidant capacity (400–500 μmol of Trolox equiv/g) among several crops (e.g., wheat, rice, oats, barley, maize, potato)^3,5^ and plant food sources of natural antioxidants^6,7^. In addition to human food, sorghum is used also for several other purposes including energy and animal nutrition^8^; it is also resilient to biotic and abiotic stresses, adapted to diverse environments, requires low agricultural inputs, all of which makes it an important crop to enhance food and commodity security across the globe^9–11^.

The *S. bicolor* genome sequence was first released in 2009^12^ and the current version 3.1.1 is sized 732.2 Megabases (Mb) and reports more than 34,000 annotated genes several of which can be used in genetic introgressions and genomic-aided improvement of yields and the quality of the products in this crop^13^. The sorghum reference genome is expected to facilitate resequencing experiments and genetic investigations in cultivated sorghum and its wild gene pool. In this work, we whole-genome resequenced and present the comparative information of *S. bicolor* and *S. bicolor* × *S. halepense* recombinant inbred lines; to our knowledge, no resequencing studies have been reported that accounted for such populations contemporarily.

Sorghum like any other crop can be genetically improved by introgressing genetic factors from wild relatives^14^. Sorghum breeders have shown interest in interspecific crosses between *Sorghum bicolor* and Johnsongrass [*Sorghum halepense* (L.) Pers.] which is a wild species natural allotetraploid (2n = 4x = 40) believed to have originated by the spontaneous hybridization between *S. bicolor* and *S. propinquum* (Kunth) Hitchc., followed by chromosome doubling^15^. Available evidence shows that Johnsongrass can confer a strong perenniality and overwintering in *S. bicolor* genetic background^14,16–19^. The ploidy of *Sorghum halepense* implies that its hybridization with *S. bicolor* requires the latter be either induced tetraploid or cytoplasmic genetic male sterile diploid; in both cases mainly tetraploid progeny is generated^19,20^, but cases of diploid descendants were observed^21,22^. The importance of introgressing perenniality in crops can be explained by the search for agroecological functions of a perennial cultivar as a long-lasting cover crop. The cover crops are environment friendly, help avoid bare soils, improve soil health, reduce agricultural inputs, foster biodiversity, which can make agricultural production more resilient to climate change adversities^23,24^. Bare soils represent one of the major failures of the conventional agricultural intensification as they cause soil and plant nutrients loss mainly through erosion and lixiviation. Perennial crops create permanent soil cover, recycle and stop loss of nutrients from fertilizers, allowing drastic reduction of rates of fertilizer application, and improving soil health. In addition, by permanently covering the soil, perennial crops limit soil moisture loss through evaporation, guarantee a high level of soil organic matter and an active soil biology, which in turn improves soil chemical and physical properties, and helps neutralize greenhouse gas emissions particularly through carbon sequestration, and hence, mitigates climate change^25^.

Like in other such studies^26^, the resequencing of sorghum populations described in this work allows capturing the natural variation across the gene pool through the identification of millions of variants among cultivated and wild *S. halepense* relative accessions. Such high-confidence polymorphisms will be used in forward genetics and linkage disequilibrium studies to unravel the genetic base of complex plant characteristics of agronomic importance, and the development of climate change resilient cultivars.

Sorghum breeders working on the introgression of perenniality in otherwise annual *S. bicolor* background select for overall sorghum bicolor plant aspect in addition to the overwintering trait. We also have observed from our experience in recent years^14^ that backcrosses are the most attractive as they show traits closely comparable to domesticated sorghum^27^ (panicle shape and compactness, bold and big seed size, absence of seed shattering, etc..) than single, double, or three-way crosses. We, therefore, used the data produced in this and previous studies from our laboratory to investigate the contribution of *sorghum halepense* in *S. bicolor* × *S. halepense* controlled hybridizations with particular interest in backcrosses involving two doses of *S. bicolor* as the recurrent parent. In this work, we describe the first whole-genome resequencing study, contemporarily evaluating *S. bicolor* and the progeny from *S. bicolor* × *S. halepense* crosses; we conducted a comprehensive structural and functional characterization of 172 lines 19 of which were recombinant inbred lines that inherited different proportions of *S. bicolor* and *S. halepense*. We interrogated the entire genome of these populations and produced a large set of robust and high-confidence variants that will sustain breeding and other genetic and genomic investigations in sorghum, including genomics-assisted breeding.

## Results

### Genetic diversity and sequencing assessment

**Figure 1:**
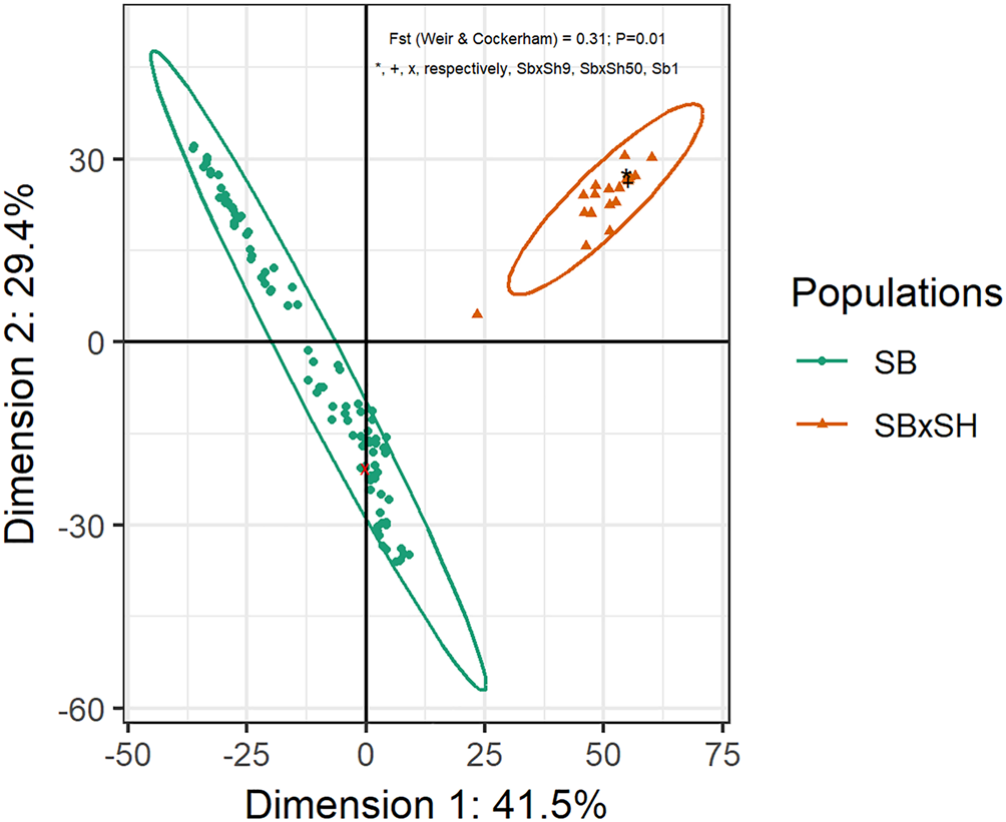
Genetic differentiation analysis in Sb and SbxSh populations. The ellipses are drawn accounting for the 95% confidence interval and the Euclidean distance from the center “o”. Genotypes outside the ellipses are outliers^74^.

One hundred and seventy-two sorghum lines evaluated in this study clustered in two different populations of 153 *S. bicolor* genotypes and 19 *S. bicolor* × *S. halepense* recombinant inbred lines (Fig. 1). The Fstatistic (Fst) measuring the genetic structure and the level of genetic differentiation^28,29^ was significantly and moderately high (Fst = 0.31, p = 0.01); Fst values range from 0 in case of panmixis to 1 in case the populations do not share any genetic diversity. The first two dimensions of the principal coordinates explained 70.9% of the total genetic diversity existing in the studied populations. The one hundred and seventy-two sorghum lines were whole-genome resequenced and a corresponding number (172) of paired-end sequencing libraries constructed each with the insert size around 300 base pairs. Resequencing yielded 22.88 billion paired-end reads resulting in 3.43 trillion bases (nucleotides) and 2.6 TB of high-quality raw data. Ultimately, a total of 21.70 billion and 3.25 trillion of clean paired-end reads and bases were produced, respectively. Overall, 94.54% of total clean reads showed quality value Q20 ≥ 94.54%, which indicates a high data quality. Sorghum bicolor (Sb) and *S. bicolor* × *S. halepense* (SbxSh) showed comparable clean reads quality (Q20 = 96.17–96.38% and Q30 = 87.91–88.22%), number of clean reads (125.7–126.19 × 10^6^) and bases (18.82–18.89 × 10^9^), and the ratio clean bases: raw bases (94.64–94.89%). However, the guanine-cytosine (GC) rate was higher in SbxSh relative to Sb (43.42% a *vs.* 43.26% b) in the clean data.

### Sequence alignment

#### Alignment assessment

Sequence reads were aligned to the Sorghum bicolor (BTx623) reference genome whose size was 732,200,000 bp, while the effective size was 675,973,270 bp (N base excluded), and GC content of 41.82%. The mapping rate i.e., the percentage coverage of the reference by reads of the sorghum samples varied from 89.15 to 95.18% with a mean of 92%. The percentage of mapped reads and that of mapped bases were identical and ranged from 82.65 to 99.92% with an average of 99.40%. The effective mapping depth i.e., LN/G, where L is the read length, N is the number of reads and G is the haploid genome length, was between 23.17X to 34.38X. In this work, SbxSh and Sb showed comparable mapped reads (99.04–99.45%), mapped bases (99.04–99.45%), sequencing depth after mapping (26.54X–26.64X), while SbxSh showed statistically significant higher percentage coverage rate (94.87a vs. 91.62b), yet lower percentage unique hit bases (81.96a vs. 77.58b) and unique hit reads (82.39a vs. 78.31b).

#### SNP calling and annotation

**Figure 2:**
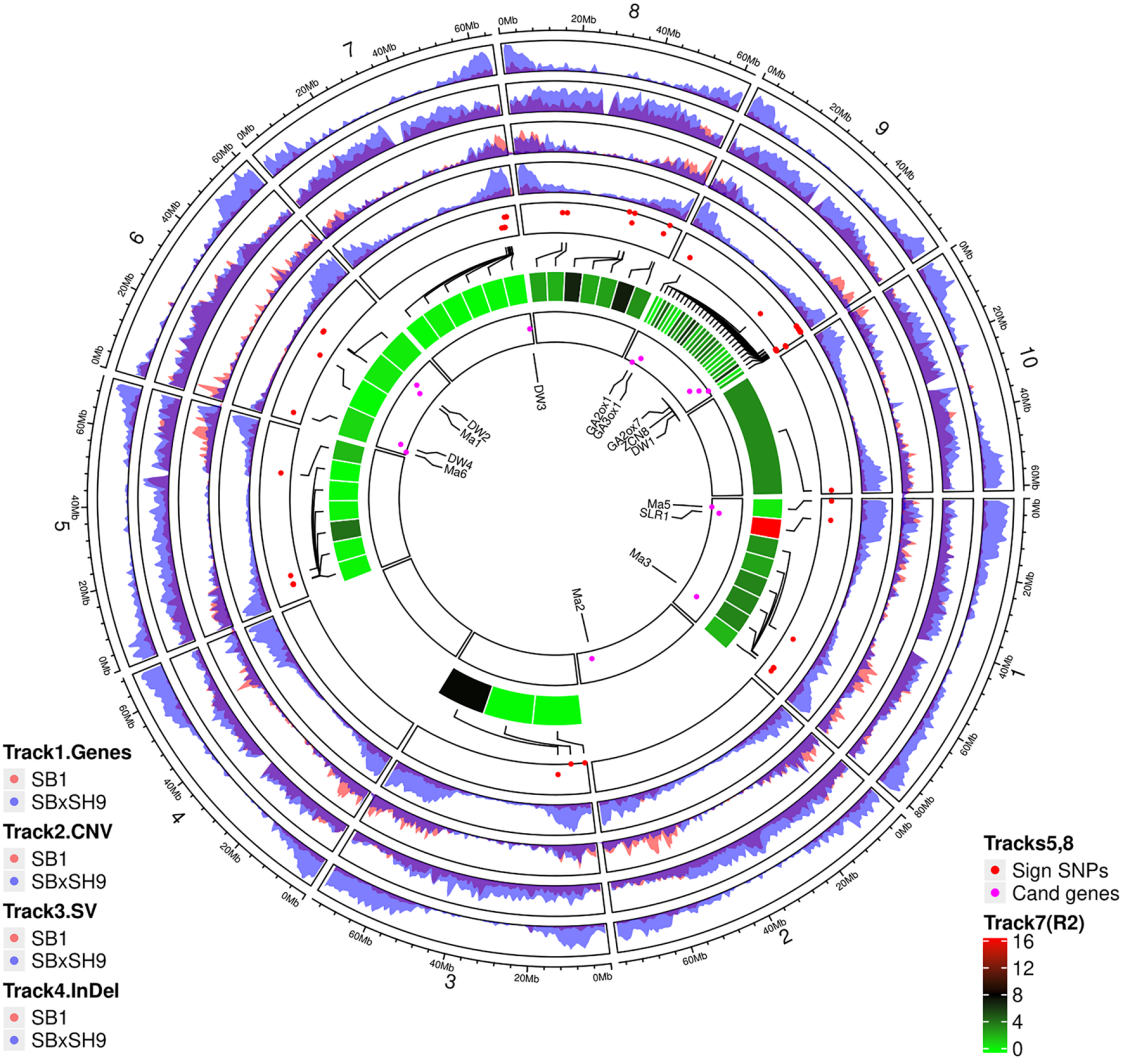
Chromosomal distribution of WGRS information in Sb and BC1 SbxSh lines, and GWAS data from the evaluated populations^74^. The x-axis corresponds to the genomic coordinate. Tracks 1–4: visualize the genomic density of regions (defined as the fraction of a genomic window that is covered by genomic regions). Tracks 5, 8: significant SNPs and candidate genes are displayed according to their genomic coordinates (x-axis), while y-values were set for the sole purpose improving the resolution (legibility) of the corresponding SNPs and candidate genes.

The sequence alignment of the target sorghum lines to the reference genome BTx623, the gene models, and the information derived from the reference genome allowed to identify large numbers of SNPs, InDels, CNVs, and SVs (Table 1; Fig. 2). A total of 567,046,841 SNPs was uncovered from these sorghum genomes. On average, 10,515,367.4 SNPs were observed per individual, of which 1,855,062 were located in the genic regions. The statistical analysis showed that SbxSh had more total and heterozygous SNPs, synonymous_CDS, nonsyn_CDS, exonic, genic, intronic, mRNA, pseudogenic, transcript, and tRNA SNPs, whereas Sb showed more homozygous SNPs. Among the synonymous and nonsynonymous SNPs mapped in the coding regions in either population, the synonymous SNPs represented 49% and 54%, respectively, in Sb and SbxSh populations. The SbxSh population contained 81% and 78%, respectively, of all synonymous and nonsynonymous SNPs. Contrary to synonymous mutations, nonsynonymous mutations cause variation in coding amino acids and are considered to play a significant role in changing the phenotype of organisms. Besides, nonsynonymous mutations are also strong candidates to explain the phenotypic diversity between different individuals in a population.

**Table 1:** Tukey HSD for SNPs, InDels, SVs, and CNVs in *S. bicolor vs. S. bicolor* × *S. halepense* results.

SNPs	Total SNPs	Homozygous	Heterozygous	Syn_CDS	Nonsyn_CDS	Exon	Gene	Intron	mRNA	Pseudogene	Transcript	tRNA
Sb-SNP	2,740,707.91 b	1,568,460.64 a	1,172,247.27 b	51,930.82 b	53,090.27 b	259,126.96 b	367,089.86 b	0.28 b	502,355.61 b	24,551.5 b	66,349.91 b	47.39 b
SbxSh-SNP	7,774,659.53 a	266,261.84 b	7,508,397.68 a	224,011.11 a	192,976.26 a	1,039,206.53 a	1,487,971.84 a	0.79 a	2,098,316.74 a	72,998.58 a	248,660 a	97.89 a
CNVs, SV, InDels	CDS	cDNA match	Direct repeat	Exon	Gene	Intron	Lnc RNA	mRNA	Pseudogene	rRNA	tRNA	Transcript
Sb-CNV	220,969.2 a	813.5 b	11.71 a	299,489.67 b	35,240.17 b	6.01 a	6870.76 b	44,166.06 b	1481.2 b	11.03 a	569.77 a	3868.22 b
SbxSh-CNV	220,873.05 a	816 a	12.42 a	299,802.58 a	35,634.95 a	6:00 AM	7426.47 a	44,819.11 a	1565 a	11.05 a	569.74 a	4134.21 a
Sb-SV	613,211.95 b	2270.12 a	6459.33 a	831,108.19 b	89,783.18 b	293.41 a	12,531.91 b	1616.44 a	3531.11 a	212.8 a	1615.27 a	5385.1 b
SbxSh-SV	714,082.79 a	2530.32 a	19.68 a	976,566.21 a	104,589.79 a	18.79 a	15,119.68 a	1739.21 a	3927.74 a	18.63 a	1640.47 a	6678.53 a
Sb-InDels	12,638.73 b	205.48 b	48,117.67 a	88,723.88 b	27,024.42 b	NA	74,829.22 b	361,489.41 b	345,870.29 a	NA	6755.33 a	1708.89 b
SbxSh-InDels	36,324.74 a	604.53 a	65,756.26 a	262,396.16 a	268,724.05 a	NA	134,405.79 a	662,402.89 a	419,384.79 a	NA	8914.84 a	20,483.42 a

*Numbers followed by same letter within a column and for same variant, are not statistically different at the 5% probability level.

**Figure 3:**
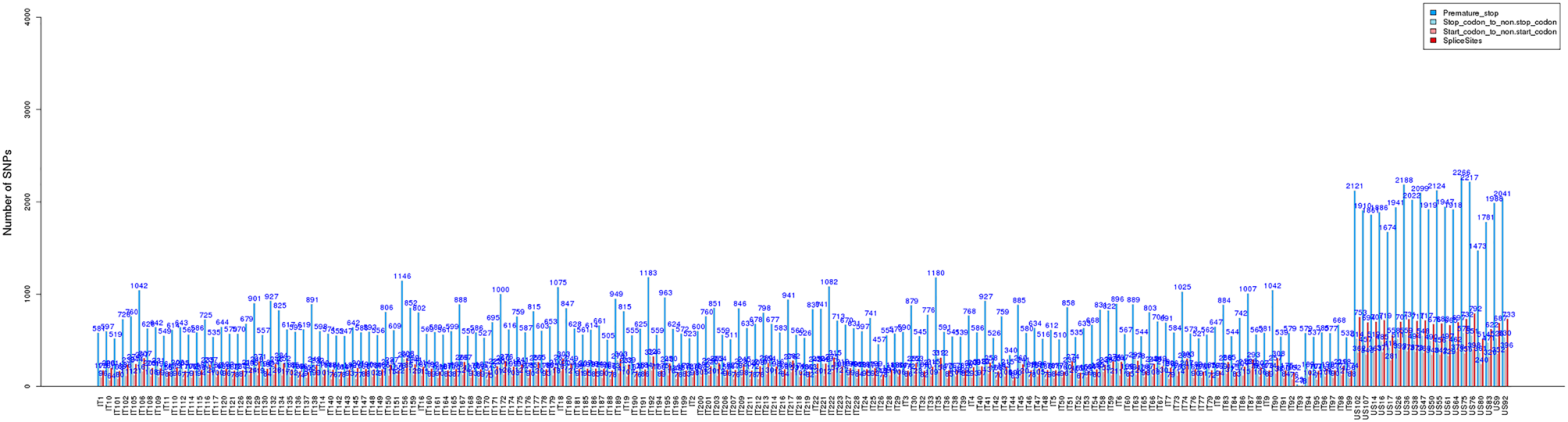
Statistics of different types of large-effect SNPs. Re-sequenced entries are represented on x-axis, numbers on top of each bar represent the number of SNPs^74^.

We further analyzed the distribution of the large-effect SNPs i.e., those with a potential to disable gene functions^26,30^. In this work, large-effect SNPS included premature stop codon, stop codon to non-stop codon, start codon to non-start codon, and splice sites. It was found that within the 10,140 SNPs participating in codon premature termination, 2,970 SNPs disrupt splicing donor or acceptor sites of genome, 13,976 SNPs are related to alteration of initiation methionine residues, and 1,144 SNPs replace terminators with certain amino acid residues that leads to longer ORFs. The statistics are depicted in Fig. 3 where SbxSh showed higher numbers of large-effect SNPs than Sb. In SbxSh population, it was found an average of 1967, 500, 370, and 700 SNPs expected to induce premature stop codon, stop codon to non-stop codon, start codon to non-start codon, and splice sites, respectively, while in Sb it was found a maximum of 1183, 340, 100, and 340, respectively. In both populations, SNPs inducing premature stop codon were most represented with respect to the other large-effect SNPs.

#### InDel calling and annotation

A total of 91,825,474 indels was identified of which 24% and 76% resided in Sb and SbxSh, respectively; mean individual insertions (211,283.62 vs. 649,218.63) and deletions (221,602.78 vs. 697,826.37) were statistically higher in SbxSh relative to Sb population. The genome-wide distribution of short InDels (1–10 bp) showed a lower number of these variants in genes and coding regions compared with Pseudogenes and mRNA, for instance (Table 1). Our result show that indels that are not multiples of 3 bp and produce frameshift mutations are particularly uncommon in coding regions. Frame shift mutation in CDS region, 3X-shift mutation in CDS region, 3X-shift mutation in CDS region phase 0, and 3X-shift mutation in CDS region phase No 0 were statistically higher in SbxSh than in Sb i.e., 16,544, 19,780.74, 6301.53, 13,479.21 vs. 5954.58, 6684.15, 2114.42, 4569.73, respectively. A frameshift mutation results from an insertion or deletion of a number of nucleotides that is not a multiple of three. The change in reading frame alters every amino acid after the point of the mutation and results in a nonfunctional protein. The comparative effects of frame-shifting (e.g., 1-, 2-, 4-, 5-, 7-, 8-, 10-bp.) non-frame-shift (e.g., 3-, 6-, 9-bp) shows that the former short InDels provide a much powerful explanation of the difference of traits between individuals^30^.

#### Structure and copy number variations

In this study, a total of 1,532,171 SVs was identified and found statistically comparably distributed between the two populations. Of the observed SVs, Sb and SbxSh showed statistically comparable individual mean numbers of deletions (4202 vs 4119), other SVs (4699 vs 4594), but SbxSh displayed more insertions than Sb, i.e., 72.47 vs 21.01 individual mean SV number. A total of 4,973,961 CNVs was generated from the entire population, with SbxSh producing statistically higher number of CNVs than Sb (41,296.21 vs. 27,381.26), higher CNV up-regulation (16,650.26 vs. 10,567.82) and down-regulation (24,214.95 vs 16,345.06).

### Genetic variation in *S. bicolor* and *S. bicolor* × *S. halepense* hybrids

One of our working hypotheses was that some of the identified genetic variation might contribute to the phenotypic differentiation between *S. bicolor* and *S. bicolor* × *S. halepense* which pushed us to focus our analysis on SNPs in genic regions. Sorghum breeders working on the introgression of perenniality in otherwise annual *S. bicolor* background select for overall *sorghum bicolor* plant aspect in addition to the overwintering trait. We also observed from our experience in recent years^14^ that backcrosses are the most attractive as they show traits closely comparable to domesticated sorghum (panicle shape, seed size, etc..) than single, double, or three-way crosses. We therefore used the data produced in this and previous studies from our laboratory to investigate the contribution of *Sorghum halepense* in SbxSh controlled hybridizations. As Fig. 2 shows, SbxSh9 backcross line has more genes and more SNPs, CNVs, and indels than *S. bicolor* Sb1; nonetheless, the two lines displayed comparable number of SVs. The same pattern was observed across populations. The density of genes, SNPs, indels, and SVs increases from the pericentromeric region towards the telomeres, with SNPs/genes and short indels showing similar distribution pattern. In both populations, the distribution of CNVs was homogeneous from the centromeres to telomeres in all chromosomes. In addition, sorghum biomass related significant SNPs and candidate genes recently uncovered^8^ in these populations i.e., *Dw* genes (*Dw1*, *Dw2*, *Dw3*, *Dw4*), *Ma* genes (*Ma1*, *Ma2*, *Ma3*, *Ma5*, *Ma6*), gibberellin (GA) associated genes (*SbGA2ox1*, *SbGA3ox1*, *SbGA2ox7*), genes involved in controlling heading date (*SbZCN8*)^1,31^ and GA signaling and plant height regulation (*SbSLR1*^1^) were mostly localized towards the distal and proximal ends of the chromosomes of interest (Fig. 2).

**Figure 4:**
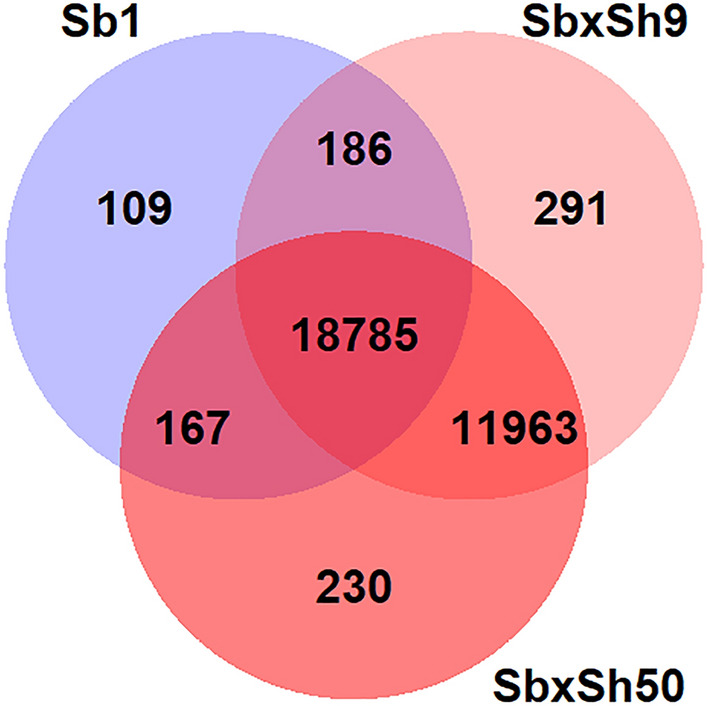
Number of shared and private genes among Sb1 (IESV 99,091 DL) and two sister perennial RILs (SbxSh9 and SbxSh50) derived from SbxSh cross backcrossed (2 recurrent parent doses: Tx623*2/Gypsum 9; BC1) to *S. bicolor*^74^.

The analysis of genes harboring SNPs showed more genes (18,785) were shared between Sb and BC1 crosses, with relatively fewer genes i.e., 109, 230, and 291 being private to the respective three lines Sb1, SbxSh50, and SbxSh9 (Fig. 4). The analysis of the biological processes (BP) GO enrichment associated to SbxSh genes showed 33,693, and 5548 genes in the Sorghum bicolor reference genome dataset and in SbxSh9/SbxSh5 hybrids gene set that mapped to the GO terms (either directly or through inheritance), respectively (Table 2). The most granular terms included enrichment in genes associated to the plant-type cell wall organization (GO:0009664), root development (GO:004836), cell wall polysaccharide metabolic process (GO:0010383), glutathione metabolic process (GO:0006749), hydrogen peroxide catabolic process (GO:0042744), anatomical structure morphogenesis (GO:0009653), response to oxidative stress (GO:0006979), proteasomal protein catabolic process (GO:0010498), response to oxygen-containing compound (GO:1901700), generation of precursor metabolites and energy (GO:0006091), translation (GO:0006412), hormone-mediated signaling pathway (GO:0009755), response to abiotic stimulus (GO:0009628), vesicle-mediated transport (GO:0,016,192), ribonucleoprotein complex biogenesis (GO:0022613), protein-containing complex subunit organization (GO:0043933), intracellular transport (GO:0046907), cellular component assembly (GO:0022607), protein localization (GO:0008104), organelle organization (GO:0006996), nitrogen compound transport (GO:0071705), organic substance transport (GO:0071702), small molecule metabolic process (GO:0044281), regulation of transcription, DNA-templated (GO:0006355), nucleobase-containing compound metabolic process (GO:0006139), positive regulation of translation (GO:0045727).

**Table 2:** Hierarchical relations between over-represented (enriched) functional classes using GO biological process annotation in private genes of S. bicolor*2/*S. halepense* relative to *S. bicolor* lines.

Biological process category^¥^	*S. bicolor*	Obs	Exp	Fold	O/U	P.value
Plant-type cell wall organization (GO:0009664)	66	28	10.87	2.58	+	2.02E−02
Cell wall organization (GO:0071555)	249	78	41.00	1.90	+	3.20E−04
Cell wall organization or biogenesis (GO:0071554)	365	104	60.10	1.73	+	3.05E−04
Cellular process (GO:0009987)	11,897	2536	1959.00	1.29	+	5.48E−54
External encapsulating structure organization (GO:0045229)	268	81	44.13	1.84	+	7.57E−04
Cellular component organization (GO:0016043)	1929	469	317.64	1.48	+	2.85E−13
Cellular component organization or biogenesis (GO:0071840)	2271	558	373.95	1.49	+	3.22E−17
Root development (GO:0048364)	87	34	14.33	2.37	+	1.36E−02
Root system development (GO:0022622)	87	34	14.33	2.37	+	1.36E−02
Anatomical structure development (GO:0048856)	645	152	106.21	1.43	+	2.87E−02
Cell wall polysaccharide metabolic process (GO:0010383)	118	43	19.43	2.21	+	5.25E−03
Cellular macromolecule metabolic process (GO:0044260)	4903	983	807.34	1.22	+	8.72E−08
Cellular metabolic process (GO:0044237)	8202	1731	1350.57	1.28	+	6.81E−28
Metabolic process (GO:0008152)	9514	1998	1566.61	1.28	+	4.27E−33
Macromolecule metabolic process (GO:0043170)	6247	1253	1028.65	1.22	+	4.80E−11
Organic substance metabolic process (GO:0071704)	8662	1785	1426.31	1.25	+	6.68E−24
Carbohydrate metabolic process (GO:0005975)	976	218	160.71	1.36	+	1.52E−02
Primary metabolic process (GO:0044238)	8162	1643	1343.98	1.22	+	6.05E−17
Glutathione metabolic process (GO:0006749)	107	38	17.62	2.16	+	3.35E−02
Peptide metabolic process (GO:000006518)	683	190	112.47	1.69	+	2.06E−08
Organonitrogen compound metabolic process (GO:1901564)	5182	1074	853.29	1.26	+	2.05E−12
Nitrogen compound metabolic process (GO:0006807)	6844	1407	1126.96	1.25	+	1.16E−16
Cellular amide metabolic process (GO:0043603)	786	210	129.43	1.62	+	5.56E−08
Cellular nitrogen compound metabolic process (GO:0034641)	2981	669	490.86	1.36	+	1.21E−12
Sulfur compound metabolic process (GO:0006790)	301	81	49.56	1.63	+	4.80E−02
Hydrogen peroxide catabolic process (GO:0042744)	162	53	26.68	1.99	+	8.69E−03
Cellular catabolic process (GO:0044248)	1250	300	205.83	1.46	+	4.10E−07
Catabolic process (GO:0009056)	1527	375	251.44	1.49	+	1.06E−10
Hydrogen peroxide metabolic process (GO:0042743)	163	54	26.84	2.01	+	5.00E−03
Reactive oxygen species metabolic process (GO:0072593)	178	58	29.31	1.98	+	3.59E−03
Anatomical structure morphogenesis (GO:0009653)	182	58	29.97	1.94	+	6.91E−03
Response to oxidative stress (GO:06979)	274	85	45.12	1.88	+	1.38E−04
Response to stress (GO:0006950)	1514	343	249.30	1.38	+	9.78E−06
Response to stimulus (GO:050896)	2823	614	464.84	1.32	+	5.24E−09
Proteasomal protein catabolic process (GO:0010498)	216	65	35.57	1.83	+	1.14E−02
Protein metabolic process (GO:0019538)	4243	836	698.67	1.20	+	6.82E−05
Organonitrogen compound catabolic process (GO:1901565)	715	165	117.73	1.40	+	3.75E−02
Organic substance catabolic process (GO:1901575)	1308	312	215.38	1.45	+	3.32E−07
Macromolecule catabolic process (GO:0009057)	842	191	138.65	1.38	+	2.31E−02
Cellular protein metabolic process (GO:0044267)	3814	761	628.03	1.21	+	4.83E−05
Response to oxygen-containing compound (GO:1901700)	368	101	60.60	1.67	+	2.31E−03
Response to chemical (GO:0042221)	1117	281	183.93	1.53	+	1.55E−08
Generation of precursor metabolites and energy (GO:0006091)	440	119	72.45	1.64	+	5.64E−04
Translation (GO:0006412)	541	146	89.08	1.64	+	3.08E−05
Peptide biosynthetic process (GO:0043043)	548	149	90.24	1.65	+	1.40E−05
Amide biosynthetic process (GO:0043604)	601	158	98.96	1.60	+	4.19E−05
Cellular nitrogen compound biosynthetic process (GO:0044271)	1258	293	207.15	1.41	+	1.14E−05
Cellular biosynthetic process (GO:0044249)	2478	558	408.04	1.37	+	2.55E−10
Biosynthetic process (GO:0009058)	2735	603	450.35	1.34	+	8.91E−10
Organonitrogen compound biosynthetic process (GO:1901566)	1278	323	210.44	1.53	+	2.32E−10
Organic substance biosynthetic process (GO:1901576)	2576	569	424.17	1.34	+	3.32E−09
Gene expression (GO:0010467)	1647	386	271.20	1.42	+	1.85E−08
Cellular macromolecule biosynthetic process (GO:0034645)	1332	309	219.33	1.41	+	6.35E−06
Macromolecule biosynthetic process (GO:0009059)	1379	316	227.07	1.39	+	1.37E−05
Hormone-mediated signaling pathway (GO:0009755)	315	85	51.87	1.64	+	2.80E−02
Cellular response to hormone stimulus (GO:0032870)	319	85	52.53	1.62	+	4.33E−02
Cellular response to organic substance (GO:0071310)	396	107	65.21	1.64	+	2.26E−03
Cellular response to chemical stimulus (GO:0070887)	677	185	111.48	1.66	+	1.53E−07
Cellular response to stimulus (GO:0051716)	1731	370	285.03	1.30	+	8.32E−04
Response to organic substance (GO:0010033)	685	182	112.79	1.61	+	1.72E−06
Response to endogenous stimulus (GO:0009719)	543	144	89.41	1.61	+	1.06E−04
Response to hormone (GO:0009725)	538	144	88.59	1.63	+	6.22E−05
Regulation of cellular process (GO:0050794)	3891	756	640.70	1.18	+	2.51E−03
Regulation of biological process (GO:0050789)	4249	809	699.65	1.16	+	1.62E−02
Biological regulation (GO:0065007)	4812	939	792.36	1.19	+	4.36E−05
Response to abiotic stimulus (GO:0009628)	505	136	83.15	1.64	+	1.06E−04
Vesicle-mediated transport (GO:0016192)	454	117	74.76	1.57	+	6.54E−03
Transport (GO:0006810)	2373	517	390.75	1.32	+	2.73E−07
Establishment of localization (GO:0051234)	2407	523	396.34	1.32	+	3.07E−07
Localization (GO:0051179)	2509	551	413.14	1.33	+	1.90E−08
Ribonucleoprotein complex biogenesis (GO:0022613)	433	111	71.30	1.56	+	1.43E−02
Cellular component biogenesis (GO:0044085)	1079	275	177.67	1.55	+	6.97E−09
Protein-containing complex subunit organization (GO:0043933)	530	130	87.27	1.49	+	2.00E−02
Intracellular transport (GO:0046907)	666	163	109.67	1.49	+	1.84E−03
Establishment of localization in cell (GO:0051649)	700	170	115.26	1.47	+	1.71E−03
Cellular localization (GO:0051641)	821	204	135.19	1.51	+	2.85E−05
Cellular component assembly (GO:0022607)	641	156	105.55	1.48	+	4.23E−03
Protein localization (GO:0008104)	736	169	121.19	1.39	+	3.86E−02
Macromolecule localization (GO:0033036)	904	211	148.86	1.42	+	1.33E−03
Organelle organization (GO:0006996)	1308	297	215.38	1.38	+	8.81E−05
Nitrogen compound transport (GO:0071705)	968	217	159.39	1.36	+	1.27E−02
Organic substance transport (GO:0071702)	1156	258	190.35	1.36	+	2.43E−03
Small molecule metabolic process (GO:0044281)	1339	292	220.48	1.32	+	3.09E−03
Regulation of transcription DNA-templated (GO:0006355)	2044	428	336.57	1.27	+	8.47E−04
Regulation of nucleic acid-templated transcription (GO:1903506)	2044	428	336.57	1.27	+	8.47E−04
Regulation of RNA biosynthetic process (GO:201141)	2044	428	336.57	1.27	+	8.47E−04
Regulation of RNA metabolic process (GO:0051252)	2141	448	352.54	1.27	+	4.82E−04
Regulation of nucleobase-containing compound m. proc. (GO:0019219)	2179	455	358.80	1.27	+	4.83E−04
Nucleobase-containing compound metabolic process (GO:0006139)	2183	449	359.46	1.25	+	2.72E−03
Organic cyclic compound metabolic process (GO:1901360)	2580	546	424.83	1.29	+	4.65E−06
Heterocycle metabolic process (GO:0046483)	2389	505	393.38	1.28	+	2.27E−05
Cellular aromatic compound metabolic process (GO:0006725)	2510	538	413.30	1.30	+	1.10E−06
Unclassified	17557	2200	2890.99	0.76	−	0.00E00
Positive regulation of translation (GO:0045727)	230	2	37.87	0.05	−	4.89E−11
Positive regulation of cellular protein metabolic process (GO:0032270)	323	24	53.19	0.45	−	1.13E−02
Positive regulation of protein metabolic process (GO:0051247)	330	24	54.34	0.44	−	5.83E−03
Positive regulation of gene expression (GO:0010628)	266	10	43.80	0.23	−	1.61E−06
Positive regulation of cellular amide metabolic process (GO:0034250)	232	2	38.20	0.05	−	3.57E−11
Regulation of cellular amide metabolic process (GO:0034248)	388	29	63.89	0.45	−	1.51E−03
Regulation of translation (GO:0006417)	383	28	63.07	0.44	−	1.06E−03
Posttranscriptional regulation of gene expression (GO:0010608)	436	34	71.79	0.47	−	9.51E−04

^¥^Sorting is done only by the most specific subclass first, with its parent terms indented directly below it. These are all related classes in an ontology, and are often interpretable as a group rather than individually. If a term is a parent of more than one term in the results table, it is shown only under its first descendant. Obs/Exp, Fold, O/U, respectively, observed/expected genes unique to SbxSh9 and SbxSh50, fold enrichment, and over-/under-representation. Analysis Type: PANTHER Overrepresentation Test (Released 20,210,224); Annotation Version and Release Date: GO Ontology database https://doi.org/10.5281/zenodo.5080993 Released 2021-07-02; Reference List: Sorghum bicolor (all 33,693 genes in database); annotation dataset: GO Biological process complete; test type: binomial; correction: Bonferroni correction for multiple testing. Displayed only results for Bonferroni-corrected P < 0.05. Of the 12,484 SbxSh9 and SbxSh50 private genes, 5563 were uniquely mapped 36 of which with multiple mapping, and 6921 unmapped, respectively.

To root development GO term (GO:004836) mapped 34 genes: GeneID:8080001, GeneID:8064680, GeneID:8054879, GeneID:8075742, GeneID:8059541, GeneID:8078975, GeneID:8081609, GeneID:8055737, GeneID:8055874, GeneID:8063307, GeneID:8058361, GeneID:8084663, GeneID:8064471, GeneID:8079326, GeneID:8079141, GeneID:8060669, GeneID:8063006, GeneID:8060622, GeneID:8080905, GeneID:8077286, GeneID:8060000, GeneID:8058075, GeneID:8074440, GeneID:8082391, GeneID:8065800, GeneID:8080849, GeneID:8085583, GeneID:8071472, GeneID:8084890, GeneID:8072111, GeneID:8063311, GeneID:8054193, GeneID:8059201, GeneID:8082281.

## Discussion

The development of perennial sorghum initiated in 2015 in our breeding program; earlier morpho-agronomic performances of evaluated materials was reported in our previous works^14^. According to our experience and available literature^19^, the production of rhizomes is the sine qua non condition for SbxSh lines to remain perennial under temperate climates like the prevalent conditions in our Italian experimental stations^14^. One of the most interesting findings from recent studies is the absence of negative trade-off between rhizome development and seed yield and aboveground biomass yield. This is expected to allow the development of high-yielding biomass, grain, and dual purpose sorghum ideotypes expressing perennating belowground structures^14,19^. *Sorghum bicolor* × *S. halepense* perennial lines showed competitiveness relative to commercial hybrids in terms of above-ground biomass production and grain yields; however mostly backcross-derived lines showed overall plant aspect^27^ and domestication traits such as high seed yield, big caryopses, seed shattering resistance, compact inflorescence and stalk strength closer to *S. bicolor* than other crosses^14,20^.

Grain yield was significantly correlated with maturity, dry mass yield, dry mass fraction of fresh material, number of culms, rhizome development, hemicellulose, and rhizome survival but important correlation coefficients were observed for maturity, number of culms and rhizome development. The traits that showed significant medium to high^32^ correlation with aboveground dry mass yield included plant height, dry mass fraction of the fresh material, number of culms, neutral detergent fiber.

In this work, we completed the first whole-genome resequencing analysis of a unique panel made up of *Sorghum bicolor* and *S. bicolor* × *S. halepense* populations. Our resequencing focused on *S. bicolor* × *S. halepense* recombinant inbred lines (RILs) instead *S. halepense *per se and this can be explained by our effort to align the resequencing experiment with our breeding program to develop perennial sorghum cultivars^14^. The SbxSh RILs were developed through crossing and selection to minimize the *S. halepense* associated linkage drag e.g., seed shattering, small-sized kernels, excessive tillers and rhizomes, and undesirable inflorescence compactness and shape in our breeding populations. The resequencing was therefore expected to explain the parental lines contributions to the genomic composition of the perennial hybrid combinations. The use of wild relatives in genetic introgressions is generally accompanied by linkage drag associated with the introduction of unfavorable traits along with the favorable ones^33^, and this necessitates a significant and time-consuming breeding effort to recover the domesticated phenotype, particularly when the primary produce is the grain^18,34^. This resequencing experiment is important in plant breeding, particularly in sorghum. Novel variants will be used for gene discovery, while a great number of uncovered high-quality polymorphisms will be harnessed in the process of genomic selection, genome-wide association studies, and marker-assisted selection. As these are founder populations for our entire breeding program, such opportunity the resequencing offered cannot be overemphasized^35^, particularly in terms of increased genomic predictions and mapping precision of quantitative trait loci. Furthermore, no genotyping platforms e.g., arrays, chips as those in use in other crop species e.g., tomato, potato or pepper, have been developed so far in sorghum for high-throughput genotyping of sorghum traits, particularly those associated with perenniality^26^. The major aim of this work was therefore to develop a large repertoire of genomic information and polymorphism data sets that can be used for gene discovery and validation, and as a source of markers to build genotyping platforms for applied breeding purposes.

In this work, a total of 21.70 billion and 3,25 trillion of clean paired-end reads and bases were produced, respectively. Overall, 94.54% of total clean reads showed quality value Q20 ≥ 94.54%; such quality value exceeded 96% when calculated in *S. bicolor* and *S. bicolor* × *S. halepense* separately, which indicates a high data quality. The mapping rate i.e., the percentage coverage of the reference by reads of the sorghum samples varied from 89.15% to 95.18% with a mean of 92%, while the percentage of mapped reads ranged from 82.65% to 99.92% with an average of 99.40%, indicating a high sequencing accuracy and the absence of contaminating DNA. The effective mapping depth was between 23.17X to 34.38X, which was largely sufficient (Zheng et al. 2011) for aligning most of the sequences of the target samples, and testified to the high quality of the reference genome. The mapping depth obtained in this study was higher than in most previous mapping experiments that showed values around 10X^36,37^, and encompassed the entire reference genome length in homogeneous pattern in all accessions. The mapping rate and the percentage of mapped reads realized in this study were better than reported in previous works, and confirm the high quality of the reference sequence used. For instance, Gramazio et al.^26^ reported a mean mapping rate of 85.4% with a range from 76.9 to 88.7%. On the other hand, in some model species, the average rates of unmapped reads were higher e.g., 3–5% in tomato^38,39^ and 10–15% in rice^40,41^. Differences in mapping experiments can be attributed to a variety of factors including differences in: (1) the progress of the sequence assembly, (2) the levels of repetitive elements, (3) genetic divergence between the sequenced samples and the reference genome, and (4) the levels of variants polymorphisms^38,41^.

*Sorghum bicolor* × *S. halepense* and *S. bicolor* populations showed comparable mapped reads, mapped bases, sequencing depth after mapping, while *Sorghum bicolor* × *S. halepense* showed statistically significant higher percentage coverage rate (94.87a vs. 91.62b), yet lower unique percentage hit bases (81.96a vs. 77.58b) and unique percentage hit reads (82.39a vs. 78.31b). Since Sorghum bicolor (Sb) and *S. bicolor* × *S. halepense* showed comparable clean reads quality (Q20 and Q30), and the number of clean reads and bases, the higher coverage rate observed in *S. bicolor* × *S. halepense* can be attributed to the lower rates of uniquely mapped reads and hence the existence of reads mapping to multiple reference genomic loci with low level of sequence similarity with the target sequence. The existence of multi-mapped reads in *S. bicolor* × *S. halepense* can be explained by this population producing statistically higher number of short indels, insertions of long fragments (at least 50 bp), CNVs, CNV up-regulations and down-regulations than *S. bicolor*^42^. A relatively lower level of sequence similarity was expected between *S. bicolor* reference sequence and *S. bicolor* × *S. halepense* in virtue of the genetic distance that existed between the two genomes (Fig. 1) deriving mainly from *S. halepense*. Under such circumstances, several authors^39^ pointed out the need for sequencing and assembly several reference genomes from crop wild relatives to avoid biased resequencing analyses and to improve the rate of uniquely mapped reads. However, since the dynamics of gene gains and losses during plants evolution and particularly during the interploid hybridization between *S. bicolor* and *S. halepense* is not yet fully understood, other reasons may explain the higher genome coverage of *S. bicolor* × *S. halepense.*

The SbxSh population showed a greater degree of heterozygosity (Table 1), which is consistent with previous genetic analyses^34^, and can be explained by the genetic history of *S. bicolor* that underwent the bottleneck of domestication, resulting in a narrowing of the genetic base with respect to wild species, while the tetraploid nature of *S. halepense* and its progeny may have played a major role in the heterozygosity observed in the SbxSh population; the fixation of alleles requires a higher number of generations in polyploids, and heterozygosity decreases slowly even in the presence of repeated cycles of self-fertilization^43^. Furthermore, the whole-genome resequencing reads were aligned to the *S. bicolor* reference genome^13^; alignment of sequences from an allotetraploid SbxSh to a diploid genome can result in an overestimation of heterozygous loci due to alignment of homeologs. In *S. halepense* homeologs derived from orthologs in the genomes of its ancestors (*S. bicolor* and *S. propinquum*) are conserved, but following *S. bicolor* × *S. halepense* hybridization it becomes difficult to predict the fate of such homeologs across generations because of different possibilities of chromosome pairing and independent assortment at meiosis^44^. It is nonetheless expected that at least some of the homeolog chromosome pairs can be maintained and contribute to increasing the heterozygosity of the recombinant inbred lines.

Our study has identified a large set of polymorphisms, consisting of 665,378,447 of high-quality variants including SNPs, indels, SVs, and CNVs; SNPs represented 85.22% of all variants, which is in agreement with previous works^26^. The identification of more SNPs in the present sorghum panel represents a good breeding opportunity as these markers are cheaper and easy to automate for high-throughput genotyping with respect to other markers^45,46^. The whole-genome resequencing completed in this work is therefore the starting point to develop a large number of markers not only in *S. bicolor* but also in Sorghum wild relatives such as *Sorghum halepense*, for which the lack of such information slowed down their use in breeding programs^39,47^. There are success stories on the opportunity to harness variants polymorphisms from crop wild relatives e.g., in soybean^37,48^, rice^41^, and tomato^39,49^, and eggplant^26^. In our study, SbxSh population produced more variants than Sb population, which confirmed the findings in previous works showing that crop wild relatives yield more variations relative to landraces or cultivated accessions. Our study highlights therefore the possibility for a controlled introgression of the variation from *S. halepense* to broaden the genetic basis of *S. bicolor*; similar introgressions were achieved in other crops e.g., rice, tomato, and wheat^50^. To our best knowledge, our study represents the first effort to harness the valuable large pool of genetic diversity from *S. halepense* using whole genome resequencing. Similar panels were evaluated in previous studies but relied on genotyping-by-sequencing platforms that showed technical limitations particularly associated with very low sequencing depth (~ 1.5X) and poor coverage^8,34^. Examples of such previous genotyping-by-sequencing-based investigations include linkage disequilibrium studies on biomass and biomass-related trait in sorghum^8^, antioxidant traits in sorghum^34^, and Sorghum plant architecture^51^. In addition, Habyarimana and Lopez used genotyping-by-sequencing SNPs to carry out genomic prediction and selection in sorghum^52,53^. The information produced in this work and the variants identified from *S. halepense* are expected to accelerate the introgression of perenniality and other useful genomic regions of this rhizome-producing species^18^ to develop superior climate/resilient and agroecological practice compliant sorghum cultivars. A large number of high-confidence polymorphisms was also identified in *S. bicolor* population and will be harnessed for high-throughput genotyping in cultivated or wild sorghum species using high-throughput genotyping platforms e.g., arrays or chips. In this work, genotypes were called individually for each sample for all variants but, for SNPs, we also performed joint genotyping across samples to produce a multi-sample VCF call-set for further investigations. The multi-sample VCF call-set produced vcf files of 33 and 6 Mb of SNPs and indels, respectively, with a good coverage and sequencing depth. These matrices will be used to provide more insights and improving previous studies particularly in domestication, genomic predictions, genome-wide association studies, and phylogenetics^26^.

The Fst statistics which is a metric of population structure, confirmed previous studies^8^ showing that Sb and SbxSh form two distinct populations. In addition, the differentiation between the two populations is supported by the higher number of variants observed in the SbxSh population, particularly SNPs, large-effect SNPs, CNVs, SVs, indels and frameshift mutations. The Fst achieved in this work was higher than or comparable to previous reports^8,54^; the observed Fst discrepancies can be attributed to differences in the number of markers used, population genetic diversity, and in the sampling approaches implemented in these works. An outlier (SbxSh102) was identified that is genetically closer to *S. bicolor* population. The SbxSh102 consists of two doses of *S. bicolor* recurrent parent (Tx623) in the *S. bicolor* × *S. halepense* (Gypsum 9) controlled cross and this perennial RIL is of future genetics and breeding interests in the development of perennial Sorghum bicolor ideotypes.

Our study produced genes and variants with a higher density that better covered entire lengths of individual chromosomes than in previous works^52,55,56^. The density of genes, SNPs and indels showed similar chromosomal distribution pattern, increasing from the pericentromeric region towards the telomeres. Such better variants distribution pattern is expected to boost novel gene and major marker discovery. In previous works^8,31,57^, uncovered significant SNPs and candidate genes were mostly localized towards the distal and proximal ends of the chromosomes of interest. Our whole-genome resequencing work produced high density of marker variants covering entire genome and offers therefore the opportunity to uncover novel major marker variants and genes in the pericentromeric regions that currently show scarce such information. The analysis of the biological processes GO enrichment associated to SbxSh genes showed 5,548 private genes that mapped to the important GO terms; 34 of these genes mapped to root system development (GO:0022622) two (GeneID:8054879 and GeneID:8072111) of which were reported to govern root properties^58,59^. Studies conducted in Arabidopsis thaliana showed that ROOT PRIMORDIUM DEFECTIVE 1* (*RPD1*;* GeneID:8054879) is required for the maintenance of active cell proliferation and plays a critical role in the development of roots^58^, while the RETARDED ROOT GROWTH (RRG, GeneID:8072111) gene is predominantly expressed in the root meristem and encodes a mitochondria-localized protein that is required for cell division in the root meristem (Xiaojing Zhou et al.). The variants and functional analyses conducted in this work showed that mining the SbxSh private variants and genes can provide insights on genetic factors controlling plant characteristics capable of boosting sorghum genetic improvement, particularly the perenniality trait that is compliant with agroecological practices, sustainable agriculture, and climate change resilience.

## Conclusions

This work generated the first whole genome map of SNPs, indels, SVs, and CNVs in a sorghum panel that includes *S. halepense genome*, which can be used as a framework for future investigations in functional genomics and genome-assisted breeding. Sorghum is the world’s fifth economically most important cereal and is a staple particularly in the semi-arid tropics of Africa and Asia, and is globally used for varied purposes including food, feed, fodder, commercial grade alcohol, as well as first, second, and third generation biofuels. The variants (SNPs, indels, SVs and CNVs) uncovered herein will boost genomic studies e.g., genomic prediction and selection, linkage and linkage disequilibrium analyses, molecular basis of several sorghum plant characteristics, all of which can build breakthroughs to achieve significant genetic gains in sorghum crop.

## Materials and methods

### Plant materials and sequence data sets

One hundred seventy-two sorghum genotypes including 19 *S. bicolor* × *S. halepense* (SbxSh) advanced inbred lines and 153 *S. bicolor* (Sb) lines were whole-genome resequenced. *Sorghum bicolor* genotypes were comprised of tropical landraces, improved lines therefrom, and temperate breeding lines. The SbxSh lines were at different levels (F_4_–F_7_) of filial progeny generated from crosses involving annual/perennial (A/P) genotypes and A/P backcrosses to annual recurrent parents (A*2/P; BC1), perennial/perennial (P/P) and annual/perennial//perennial (A/P//P) hybrid combinations followed by cycles of selection. The annual parental lines were standard diploid (2n = 20), induced tetraploids (2n = 40), cytoplasmic-genetic male-sterile, and genetic male-sterile inbred sorghum lines. Perennial parental lines consisted of a *S. halepense* plant and tetraploid lines derived from controlled hybridization of induced tetraploid sorghum plants with *S. halepense*. These populations were described in Habyarimana et al.^8,14^. Interspecific hybridization techniques between Sb and Sh were recently described by Hodnett et al.^60^.

Total genomic DNA was extracted from 10-day old etiolated sorghum seedlings grown under standard glasshouse conditions, according to the cetyl trimethylammonium bromide method^61^, with minor modifications. DNA integrity was evaluated with agarose gel electrophoresis, DNA quality assessed through the 260/280 and 260/230 nm ratios from NanoDrop ND-1000 spectrophotometer (NanoDrop Technologies, Wilmington, Delaware, USA) and concentration measured with a Qubit® 2.0 Fluorometer (Thermo Fisher Scientific, Waltham, MA, USA). High-quality DNA samples were shipped to BGI Tech Solutions (Hongkong) Co., Limited for libraries construction and whole-genome resequencing. Paired-end libraries were prepared with an insert size of approximately 300 bp and sequenced on Illumina platform DNB-SEQ PE150 according to the supplier’s protocol, producing 20X sequencing depth resulting in 15 G bases per sample. The BTx623 sorghum reference genome sequences were downloaded from the Joint Genome Institute Phytozome website^62,63^.

### Bioinformatics analyses

#### Sequence processing, mapping, and polymorphisms calling

The raw sequences were processed with the supplier’s in-house SOAPnuke filter to obtain clean reads by discarding reads with more than 50% adaptor sequence, low quality reads for which more than 50% bases display Phred score less than 20, and reads with 2% or more “N” bases i.e., any base. The processed reads were then mapped to the reference genome of *S. bicolor* (BTx623) version 3.1.1^13^ using Burrows-Wheeler Aligner (BWA)^64^. BWA showed good performance aligning relatively short nucleotide sequences against a long reference and producing accurate and fast results with low error rates. It provides flexible parameter setup and the output of the alignment is presented in SAM format^65^. Picard-tools (v1.118)^66^ is used to sort the SAM files by coordinate, converted them to BAM files, and mark duplicated reads to be discarded by the Genome Analysis Toolkit (GATK) during downstream analyses. BAM files were further processed for mate-pair information repairing, read group information adding, and duplicate reads labeling; such postprocessed BAM files are readily used for variation detection. Single Nucleotide Polymorphisms (SNPs) and small Insertion/Deletions (InDels) are detected by GATK^67^, BreakDancer^68^ is used for Structure Variants (SVs) calling and SOAPcnv^69^ for Copy Number Variants (CNVs) calling. Genotypes were called individually for each sample for all variants but, in addition, for SNPs, we also performed joint genotyping across samples to produce a multi-sample VCF (Variant Call Format) call-set for future genomic predictions and linkage disequilibrium analyses.

#### SNP calling and annotation

To detect high-quality SNPs we first calculated the likelihood of each sample’s genotype using SOAPsnp^70^ and the genotype with the highest probability was selected as the genotype of the sequenced individual at the specific locus. Next, we selected a polymorphic locus against the reference sequence using the target consensus sequence, and based on the resequencing data of 172 samples, we determined SNPs located in effective sites with sufficient quality i.e., responding to the following criteria: 3 ≤ depth ≤ 50, with depth calculated using data from each individual, average mappable sites < 1.5, and an average quality for the novel allele > 20. The SNPs were localized in splice sites, start codons, stop codons, coding and noncoding regions, and other nucleic acid molecules based on annotated gene models in *S. bicolor* genome reference database^13^.

#### Short InDel detection

To identify short indel we mapped the paired-end reads to the reference sequence allowing up to 10-bp gaps, merged these redundant pairs, and gaps that were supported by at least three non-redundant paired-end reads were extracted. A potential indel was identified when the number of the un-gapped reads that crossed a potential indel was no more than twice that of the gapped reads. The final high-quality indels included only those identified on both strands by paired-end reads.

#### Structure variation detection

Structure variation (SV) includes deletion, insertion, duplication, inversion and transposition of long fragment (at least 50 bp) in genome. In this study, we used SOAPsv^64^ to detect SVs based on the principle of paired-end^64^ i.e., that one of the two reads of paired-end should align onto the forward chain, while the other should be aligned onto the negative (reverse) chain. In addition, the distance between the two reads after the alignment should amount the size of the insert, and pairs of two reads should have a normal orientation and a suitable span when aligned to the genome. Should the orientation or span of pairs of two reads be not consistent with alignment expectations, structural variations may be involved in that region. The abnormal paired-end alignments are analyzed by clustering and the result compared with predefined SV types. A threshold of 3 abnormal paired end reads is required to support the SV existence, while SVs that were supported by at least six paired end reads were considered of high quality and identified as the final SVs in this work.

#### Copy number variation detection

We detected CNVs by the following steps: (i) DNA sequences were separated into fragments according to the depth of each base from the alignment results; (ii) we calculated the P-value for each fragment to estimate its probability to be a CNV; and (iii) fragments that passed the criteria (fragment length longer than 2 kb, Pvalue ≤ 0.35, mean depth less than 0.5 or more than 2.0) were kept as CNVs. The P-value was calculated as the probability of each observed depth (d) under the distribution of a simulated Poisson distributed data set whose expected value (E(d)) equals the observed mean depth. If d < E(d), the P-value = P(x ≤ d) × 2, else P-value = P(x ≥ d) × 2. The credibility of a CNV is inversely proportional to the P-value.

#### *Sorghum halepense*-associated genes and gene ontology (GO) enrichment analysis

Gene ontology and in-depth molecular genetic investigation were carried out on one dual purpose Sb line derived from an improved tropical variety (IESV 99091 DL) and two sister perennial RILs (SbxSh9 and SbxSh50) derived from rhizome-growing SbxSh cross backcrossed (2 recurrent parent doses: Tx623*2/Gypsum 9; BC1) to cytoplasmic male sterile recurrent Sb parent, at more than six generations of selfing. A set of 12,484 single nucleotide polymorphism-containing genes identified in the two sister SbxSh RILs but not in IESV 99091 DL were selected as the candidate gene set associated with S. halepense (Fig. 4). These genes were mapped to gene ontology (GO)^26^ to evaluate their characteristics, using PANTHER Gene List Analysis tools^71^. PANTHER takes a set of genes and compares the frequency of GO terms in the sample set with the frequency of the same set of GO terms in the reference set to identify terms that are over- or underrepresented in the sample set. In this work, we conducted PANTHER overrepresentation test using the GO Ontology database https://doi.org/10.5281/zenodo.4735677 released May 01, 2021. The reference list consisted of Sorghum bicolor (all genes in database), while the annotation data sets were “GO molecular function complete”, “GO biological process complete”, and “GO cellular component complete”, which are the datasets with the complete, up to date GO annotations. The binomial test^72^ was used and the Bonferroni correction applied to account for multiple testing (one for each pathway, or each ontology term) at the same time. Only Bonferroni-corrected results with a probability level *P* < 0.05, were considered significant i.e., the lower the *P* value, the less likely the obtained result can be explained by random distribution.

#### Genomic mapping of biomass yield and biomass relevant traits loci in the evaluated population

The information from Habyarimana et al.^8^ was used in this work for genomic physical mapping of biomass related SNPs, candidate genes, and genes known to underpin sorghum plant height and maturity. The transcripts of known genes were identified on phytozome^63^. In their work Habyarimana et al.^8^, genome-wide association study was performed using the statistical genetics package Genome Association and Prediction Integrated Tool (GAPIT)^73^ within the R environment^74^. In addition, two multi-locus GWAS algorithms were used to identify significant quantitative trait loci (QTLs) for the biomass-related traits: BLINK (Bayesian-information and Linkage disequilibrium Iteratively Nested Keyway)^75,76^ and SUPER (Settlement of MLM Under Progressively Exclusive Relationship)^77^.

### Statistics

Statistical inferences to separate means e.g., sequencing statistics, were carried out using analysis of variance and Tukey HSD test at the 5% significance level. Genetic diversity was evaluated using the Fstatistic and the principal coordinates analysis^78^. Statistical inferences and data visualization were carried out using R software^74^.

## Data Availability

The datasets generated during and/or analyzed during the current study are not publicly available due to planned near future use, but are available from the first corresponding author on reasonable request.

## References

[1] Ordonio R, Ito Y, Morinaka Y, Sazuka T, Matsuoka M, Jeon KW (2016). Chapter Five: Molecular breeding of *Sorghum bicolor*, a novel energy crop. International Review of Cell and Molecular Biology.

[2] *ICRISAT strategic plan to 2020: Inclusive Market-Oriented Development for Smallholder Farmers in the Tropical Drylands*. (International Crops Research Institute for the Semi-Arid Tropics, 2010).

[3] Alfieri M, Balconi C, Cabassi G, Habyarimana E, Redaelli R (2017). Antioxidant activity in a set of sorghum landraces and breeding lines. Maydica.

[4] Bekele EK, Nosworthy MG, Tyler RT, Henry CJ (2021). Antioxidant capacity and total phenolics content of direct-expanded chickpea–sorghum snacks. J. Food Process. Preserv..

[5] Dykes L (2007). Phenolic compounds in cereal grains and their health benefits. Cereal Food World.

[6] Dicko MH, Gruppen H, Traore AS, van Berkel WJH, Voragen AGJ (2005). Evaluation of the effect of germination on phenolic compounds and antioxidant activities in Sorghum varieties. J. Agric. Food Chem..

[7] Wu Y (2012). Presence of tannins in sorghum grains is conditioned by different natural alleles of Tannin1. Proc. Natl. Acad. Sci. USA.

[8] Habyarimana E, De Franceschi P, Ercisli S, Baloch FS, Dall’Agata M (2020). Genome-wide association study for biomass related traits in a panel of *Sorghum bicolor* and *S. bicolor* × *S. halepense* populations. Front. Plant Sci..

[9] Awika JM, Rooney LW (2004). Sorghum phytochemicals and their potential impact on human health. Phytochemistry.

[10] Dykes L (2019). Sorghum phytochemicals and their potential impact on human health. Methods Mol. Biol..

[11] Przybylska-Balcerek A, Frankowski J, Stuper-Szablewska K (2019). Bioactive compounds in sorghum. Eur. Food Res. Technol..

[12] Paterson AH (2009). The Sorghum bicolor genome and the diversification of grasses. Nature.

[13] McCormick RF (2018). The Sorghum bicolor reference genome: Improved assembly, gene annotations, a transcriptome atlas, and signatures of genome organization. Plant J..

[14] Habyarimana E (2018). Towards a perennial biomass sorghum crop: A comparative investigation of biomass yields and overwintering of *Sorghum bicolor* x *S. halepense* lines relative to long term *S. bicolor* trials in northern Italy. Biomass Bioenergy.

[15] Paterson AH (2008). Genomics of Sorghum. Int. J. Plant Genom..

[16] Cox TS (2002). Breeding perennial grain crops. Crit. Rev. Plant Sci..

[17] Cox TS (2010). Progress in breeding perennial grains. Crop Pasture Sci..

[18] Cox S, Nabukalu P, Paterson AH, Kong W, Nakasagga S (2018). Development of perennial grain Sorghum. Sustainability.

[19] Piper J, Kulakow P (2011). Seed yield and biomass allocation in *Sorghum bicolor* and F1 and backcross generations of *S bicolor* X *S. halepense* hybrids. Can. J. Bot..

[20] Nabukalu P, Cox TS (2016). Response to selection in the initial stages of a perennial sorghum breeding program. Euphytica.

[21] Cox S (2018). High proportion of diploid hybrids produced by interspecific diploid × tetraploid Sorghum hybridization. Genet. Resour. Crop Evol..

[22] Dweikat I (2005). A diploid, interspecific, fertile hybrid from cultivated sorghum, *Sorghum bicolor*, and the common johnsongrass weed *Sorghum halepense*. Mol. Breed..

[23] Batello C (2014). Perennial Crops for FOOD Security.

[24] Hallam A, Anderson IC, Buxton DR (2001). Comparative economic analysis of perennial, annual, and intercrops for biomass production. Biomass Bioenergy.

[25] Moore KJ (2019). Regenerating agricultural landscapes with perennial groundcover for intensive crop production. Agronomy.

[26] Gramazio P (2019). Whole-genome resequencing of seven eggplant (*Solanum melongena*) and one wild relative (*S. incanum*) accessions provides new insights and breeding tools for eggplant enhancement. Front. Plant Sci..

[27] IBPGR and ICRISAT. *Descriptors for sorghum [Sorghum bicolor (L.) Moench]*. (International Board for Plant Genetic Resources, 1993).

[28] Wright S (1951). The genetical structure of populations. Ann. Eugen.

[29] Wright S (1965). The interpretation of population structure by F-statistics with special regard to systems of mating. Evolution.

[30] Zheng L-Y (2011). Genome-wide patterns of genetic variation in sweet and grain sorghum (*Sorghum bicolor*). Genome Biol..

[31] Ordonio RL (2014). Gibberellin deficiency pleiotropically induces culm bending in sorghum: An insight into sorghum semi-dwarf breeding. Sci. Rep..

[32] Gomez KA, Gomez AA (1984). Statistical Procedures for Agricultural Research.

[33] Singh M, Kumar S (2016). Broadening the genetic base of grain cereals. Springer India.

[34] Habyarimana E, Dall’Agata M, De Franceschi P, Baloch FS (2019). Genome-wide association mapping of total antioxidant capacity, phenols, tannins, and flavonoids in a panel of *Sorghum bicolor* and *S. bicolor* × *S. halepense* populations using multi-locus models. PLoS ONE.

[35] Pascual L (2015). Potential of a tomato MAGIC population to decipher the genetic control of quantitative traits and detect causal variants in the resequencing era. Plant Biotechnol. J..

[36] Guo S (2013). The draft genome of watermelon (Citrullus lanatus) and resequencing of 20 diverse accessions. Nat. Genet..

[37] Zhou Z (2015). Resequencing 302 wild and cultivated accessions identifies genes related to domestication and improvement in soybean. Nat. Biotechnol..

[38] Causse M (2013). Whole genome resequencing in tomato reveals variation associated with introgression and breeding events. BMC Genom..

[39] Aflitos S (2014). Exploring genetic variation in the tomato (Solanum section Lycopersicon) clade by whole-genome sequencing. Plant J..

[40] Subbaiyan GK (2012). Genome-wide DNA polymorphisms in elite indica rice inbreds discovered by whole-genome sequencing. Plant Biotechnol. J..

[41] Xu X (2012). Resequencing 50 accessions of cultivated and wild rice yields markers for identifying agronomically important genes. Nat. Biotechnol..

[42] Deschamps-Francoeur G, Simoneau J, Scott MS (2020). Handling multi-mapped reads in RNA-seq. Comput. Struct. Biotechnol. J..

[43] Kellogg EA (2001). Evolutionary History of the Grasses1. Plant Physiol..

[44] Rakshit S, Ganapathy KN, Visarada K (2016). Cytogenetics of Sorghum. Crit. Rev. Plant Sci..

[45] Kim C (2016). Application of genotyping by sequencing technology to a variety of crop breeding programs. Plant Sci..

[46] Yan J (2010). High-throughput SNP genotyping with the GoldenGate assay in maize. Mol. Breed..

[47] Brozynska M, Furtado A, Henry RJ (2016). Genomics of crop wild relatives: Expanding the gene pool for crop improvement. Plant Biotechnol. J..

[48] Lam H-M (2010). Resequencing of 31 wild and cultivated soybean genomes identifies patterns of genetic diversity and selection. Nat. Genet..

[49] Gao L (2019). The tomato pan-genome uncovers new genes and a rare allele regulating fruit flavor. Nat. Genet..

[50] Dempewolf H (2017). Past and future use of wild relatives in crop breeding. Crop Sci..

[51] Kong W (2021). Quantitative trait mapping of plant architecture in two BC1F2 populations of *Sorghum bicolor* × *S. halepense* and comparisons to two other sorghum populations. Theor. Appl. Genet..

[52] Habyarimana E, Lopez-Cruz M (2019). Genomic selection for antioxidant production in a panel of *Sorghum bicolor* and *S. bicolor* × *S. halepense* Lines. Genes.

[53] Habyarimana E, Lopez-Cruz M, Baloch FS (2020). Genomic selection for optimum index with dry biomass yield, dry mass fraction of fresh material, and plant height in biomass Sorghum. Genes.

[54] McClean PE (2012). Population structure and genetic differentiation among the USDA common bean (*Phaseolus vulgaris* L.) core collection. Genet. Resour. Crop Evol..

[55] Rhodes DH (2014). Genome-wide association study of grain polyphenol concentrations in global sorghum [*Sorghum bicolor* (L.) Moench] germplasm. J. Agric. Food Chem..

[56] Rhodes D, Gadgil P, Perumal R, Tesso T, Herald TJ (2017). Natural variation and genome-wide association study of antioxidants in a diverse Sorghum collection. Cereal Chem. J..

[57] Ordonio R, Ito Y, Morinaka Y, Sazuka T, Matsuoka M (2016). Molecular breeding of *Sorghum bicolor*, a novel energy crop. Int. Rev. Cell Mol. Biol..

[58] Konishi M, Sugiyama M (2006). A novel plant-specific family gene, ROOT PRIMORDIUM DEFECTIVE 1, is required for the maintenance of active cell proliferation. Plant Physiol..

[59] Zhou X (2011). The Arabidopsis RETARDED ROOT GROWTH gene encodes a mitochondria-localized protein that is required for cell division in the root meristem1[W]. Plant Physiol..

[60] Hodnett GL, Ohadi S, Pugh NA, Bagavathiannan MV, Rooney WL (2019). *Sorghum bicolor* x *S. halepense* interspecific hybridization is influenced by the frequency of 2n gametes in *S. bicolor*. Sci. Rep..

[61] Tiwari KL, Jadhav SK, Gupta S (2012). Modified CTAB technique for isolation of DNA from some medicinal plants. Res. J. Med. Plant.

[62] Phytozome info: *S. bicolor* v3.1.1. https://phytozome-next.jgi.doe.gov/info/Sbicolor_v3_1_1.

[63] Goodstein DM (2012). Phytozome: A comparative platform for green plant genomics. Nucleic Acids Res..

[64] Li H, Durbin R (2009). Fast and accurate short read alignment with Burrows-Wheeler transform. Bioinformatics.

[65] SAM/BAM/CRAM Format. *NGS Analysis* https://learn.gencore.bio.nyu.edu/ngs-file-formats/sambam-format/ (2017).

[66] Picard Tools: By Broad Institute. http://broadinstitute.github.io/picard/.

[67] Li H (2009). The Sequence Alignment/Map format and SAMtools. Bioinformatics.

[68] McKenna A (2010). The Genome Analysis Toolkit: A MapReduce framework for analyzing next-generation DNA sequencing data. Genome Res..

[69] Chen K (2009). BreakDancer: An algorithm for high-resolution mapping of genomic structural variation. Nat. Methods.

[70] Li R (2009). SNP detection for massively parallel whole-genome resequencing. Genome Res..

[71] Murugesan S, Goldberg EB, Dou E, Brown WJ (2013). Identification of diverse lipid droplet targeting motifs in the PNPLA family of triglyceride lipases. PLoS ONE.

[72] Mi H, Muruganujan A, Casagrande JT, Thomas PD (2013). Large-scale gene function analysis with the PANTHER classification system. Nat. Protoc..

[73] Tang Y (2016). GAPIT Version 2: An enhanced integrated tool for genomic association and prediction. Plant Genome.

[74] Team, R. C. *R: A Language and Environment for Statistical Computing* (2014).

[75] Liu L (2016). Original Research: A case-control genome-wide association study identifies genetic modifiers of fetal hemoglobin in sickle cell disease. Exp. Biol. Med..

[76] Huang Y-F, Poland JA, Wight CP, Jackson EW, Tinker NA (2014). Using genotyping-by-sequencing (GBS) for genomic discovery in cultivated oat. PLoS ONE.

[77] Wang Q, Tian F, Pan Y, Buckler ES, Zhang Z (2014). A SUPER powerful method for genome wide association study. PLoS ONE.

[78] Gower JC (1966). Some distance properties of latent root and vector methods used in multivariate analysis. Biometrika.

